# Skin and Genito-Urinary Diseases

**Published:** 1893-03

**Authors:** 


### SKIN AND GENITO-URINARY DISEASES.

EDITED BY DR. M. B. HUTCHINS.

### PSORIASIS.

This disease is held by dermatologists to be one which may be removed by appropriate local treatment but is almost sure to recur. Hence the common prognosis is that it is removable but not curable.

Dr. Ludwig Nielsen, of Copenhagen, has an article in *Monatshefte für Praktische Dermatologie*, Band VIII, No. 7–8, in which he advances some very plausible reasons why the disease should be considered parasitic, though the specific organism has not been discovered. He thinks the idea of heredity has had much to do with preventing the acceptance of the parasitic theory. He mentions some chrysobarin cases of the disease which he thinks illustrate contagion. Chrysobarin (chrysophanic acid) and the tars are believed the best local remedies. Internal remedies useful chiefly in rendering the skin better able to resist the parasite.

Recurrences might be attributed to reinfection from the clothing or bedding or to the remaining of one or more small, unobserved spots at the time the patient is discharged as cured. He advises thorough treatment of the disease, entire disinfection of clothing and bedding and keeping the patient under observation and the use of a mild antiparasitic ointment for some weeks, to destroy all source of reinfection.

Dr. Nielsen compares the disease to that mild, superficial epidermic disease, pityriasis versicolor, which any one who has treated it knows is almost impossible to cure because of our inability to get rid of all the fungus, even a single infected scale or thread of the clothing being sufficient to reinfect the receptive skin.

### Excision and Suture for Buboes.

Dr. Francis Sedgewick Watson, of Boston, has a paper in the February *Journal of Cutaneous and Genito-Urinary Diseases* on the "*treatment of bubo, an excision, and the attempt to secure union by first intention.*" He had treated twenty cases by this method. The list includes buboes of syphilitic, chancroidal, gonorrhœal, tubercular and traumatic nature. Following rules were followed:

1. Thoroughly remove all diseased tissue.
2. Excise all necrotic, or threatening to become necrotic, skin.
3. Curette the under surface of the skin flaps.
4. Thoroughly swab wound with dry, sterilized gauze or with solution of bichloride, 1–4000.

Care necessary to avoid wounding the cord and femoral vessels, or an obscure hernia. Enlarged lymphatic vessels must be tied to prevent lymphatic filling of cavity. Incisions were modified according to involvement of skin. He says his method, when successful, has the advantage over incising and curetting suppurating buboes that you can discharge your patient well at the end of two weeks, instead of three weeks to two months. The ten cases in which he got first intention averaged sixteen days in hospital, the remainder averaged thirty-four days. Advises the use of weak antiseptics in the wounds, or sterilization. Be sure edges are dry before suturing. Dress with large, dry, sterilized gauze dressing.

---

### How to Remove Aniline Stains From the Hands.

The stains used in microscopic work are sure to leave their mark on the hands of the operator, and those who use pyoktanin in ordinary practice seldom escape the evidence of their manipulations. A little alcohol or hydrochloric acid will generally remove the greater part of these dyes, but to do it completely some bleaching agent is required. Sodium hypochlorite, in the form of Labarraque's solution, or that of the calcium salt, are quite effective, but leaves behind the very disagreeable odor of these compounds. Unna has lately recommended a method which is convenient and unobjectionable. The hands are first washed in a solution containing a little—say five per cent.—of common salt, and then in hydrogen peroxide solution of about the same strength, being finally wiped with a cloth moistened with alcohol.—*Canada Lancet.*

The above is reproduced in the New York *Medical Record* and must be efficacious. As a rule alkalies, as in soap, only intensify these stains, while acids tend to remove them. The writer once had considerable experience with the blue pyoktanin, which was being used on an epithelioma. Water caused the powder to become a stain; soap and water intensified it. Scrubbing the hands in a basin of ordinary vinegar would usually remove the stain.

---

### KERATOSIS OF THE PALMS AND SOLES, PROBABLY DUE TO ARSENIC.

T. Colcott Fox reports in February *British Journal of Dermatology* the case of a man of twenty whose palms and soles were greatly thickened; face "muddy-looking," neck rough and dirty-looking, "body much mottled with dirty pigmentation," thought also due to arsenic. In the general, horny thickening of the palms and soles a number of flat, warty lesions could be seen, and similar ones on flexures of wrists and dorsal aspects of joints of fingers and toes. Patient had taken considerable arsenic over quite a long period, and this trouble appeared in not quite six months after beginning the arsenic. The writer believed the whole trouble due to arsenic. He had the palms and soles daily soaked in hot water and soda, then vigorously rubbed with pumice stone. Unna's strong salicylic acid plasters were kept on in the interim. This treatment resulted in a cure. He quotes authorities to prove the correctness of his opinion. So it seems arsenic not only may cure some pathological thickenings, but may set up one.

### Silver Nitrate in Gonorrhœal Epididymitis.

According to Dr. T. Trzcinski, physician to the Hôpital Saint-Lazare, at Warsaw, blenorrhagic orchi-epididymitis may be aborted in the beginning by means of local revulsion with a 1 : 10 ointment of silver nitrate. He completely envelops the diseased testicle with a small linen compress spread with a pretty thick layer of this salve; then he places cotton over the compress, applies a suspensory, and leaves the dressing in situ for twenty-four hours which the patient should pass in bed.

It is stated that the application of the ointment provokes an intense burning sensation, which, however, dies out in an hour or two; but, at the same time, the testicular pain considerably diminishes. Twenty-four hours afterward, when the dressing is removed, the scrotal skin is found partly colored black and partly red and moist, and it is observed that the testicle has become much less painful. A simple dressing of cotton is then applied for twenty-four hours, and after that the patient wears a padded suspensory.—*Merck's Bulletin.*

---

### Gonorrhœal Rheumatism.

The best treatment I have ever used for gonorrhœal rheumatism is wrapping the joint with a cloth saturated with a solution of bichloride of mercury, four grains to the ounce, and surrounding the cloth with good silk.

I am skeptical about internal treatment. I have tried protoiodide of mercury, potassium iodide and all the anti-syphilitics. Many cases lasted two or three months ; now the intolerable pain and swelling begins to subside in a few days, and the recovery is prompt.—*C. B. Hutchins, M. D., in Medical World.*

---

### Syphilis in Breach of Promise Suits.

In notes on syphilis, the above quoted journal says that syphilis constitutes a successful defense in breach of promise suits in the States of Kentucky and North Carolina.

